# Analysis of Transcriptomic Response to SO_2_ by Oenococcus oeni Growing in Continuous Culture

**DOI:** 10.1128/Spectrum.01154-21

**Published:** 2021-10-06

**Authors:** Cristobal A. Onetto, Peter J. Costello, Radka Kolouchova, Charlotte Jordans, Jane McCarthy, Simon A. Schmidt

**Affiliations:** a The Australian Wine Research Institute, Glen Osmond, South Australia, Australia; Griffith University

**Keywords:** *Oenococcus oeni*, malolactic fermentation, stress response, sulfur dioxide, transcriptomics, wine microbiology

## Abstract

To successfully complete malolactic fermentation (MLF), Oenococcus oeni must overcome wine stress conditions of low pH, high ethanol, and the presence of SO_2_. Failure to complete MLF may result in detrimental effects to the quality and stability of the resulting wines. Research efforts to date have focused on elucidating the mechanisms and genetic features that confer the ability to withstand low pH and high ethanol concentrations on O. oeni; however, the responses to SO_2_ stress are less well defined. This study focused on characterizing the transcriptional response of O. oeni to SO_2_ challenge during cultivation in a continuous system at wine-like pH (3.5). This experimental design allowed the precise discrimination of transcriptional changes linked to SO_2_ stress from responses associated with growth stage and cultivation parameters. Differential gene expression analysis revealed major transcriptional changes following SO_2_ exposure and suggested that this compound primarily interacts with intracellular proteins, DNA, and the cell envelope of O. oeni. The molecular chaperone *hsp20*, which has a demonstrated function in the heat, ethanol, and acid stress response, was highly upregulated, confirming its additional role in the response of this species to SO_2_ stress. This work also reports the first nanopore-based complete genome assemblies for O. oeni.

**IMPORTANCE** Malolactic fermentation is an indispensable step in the elaboration of most wines and is generally performed by Oenococcus oeni, a Gram-positive heterofermentative lactic acid bacterium species. While O. oeni is tolerant to many of the wine stresses, including low pH and high ethanol concentrations, it has high sensitivity to SO_2_, an antiseptic and antioxidant compound regularly used in winemaking. Understanding the physiological changes induced in O. oeni by SO_2_ stress is essential for the development of more robust starter cultures and methods for their use. This study describes the main transcriptional changes induced by SO_2_ stress in the wine bacterium O. oeni and provides foundational understanding on how this compound interacts with the cellular components and the induced protective mechanisms of this species.

## INTRODUCTION

Malolactic fermentation (MLF) is defined as the decarboxylation of l-malic acid into l-lactic acid and CO_2_ (1). It is considered an indispensable step in elaborating most wines due to the chemical changes associated with this process, including reduction of acidity, enhancement of organoleptic properties, and increased microbiological stability (2). In wine, MLF is generally performed by Oenococcus oeni, a Gram-positive heterofermentative lactic acid bacterium (LAB) species (3). MLF can occur spontaneously via the action of indigenous O. oeni; however, inoculation of selected O. oeni starter cultures is often recommended to reduce processing times and minimize the growth of spoilage microorganisms. In addition to removing potential carbon sources through MLF, winemakers also manage the risk of microbial spoilage through pH control, often mediated using tartaric acid addition, and through the addition of SO_2_, an antiseptic and antioxidant compound with a long history in winemaking (2). While O. oeni is more tolerant than many of their competitor microorganisms to low pH (4), it also has its limitations, specifically, its apparent sensitivity to SO_2_ (5).

Understanding the physiological changes induced in O. oeni under stressful wine conditions (low pH, high ethanol, and SO_2_) is essential for the development of more robust starter cultures and methods for their use. Studies focused on understanding the response of O. oeni to low extracellular pH have shown that malic acid utilization and the consequent consumption of protons creates a membrane potential that powers ATP generation via membrane-bound ATPases (6–8). Thus, low pH environments favor the survival of O. oeni as long as malic acid is present and suggests approaches that might be used to optimize the efficient initiation of MLF. The physiological responses linked to acid stress have been described and include rigidification of the plasma membrane, which appears to be irreversible over the short term (9, 10). It has been suggested that changes to the state of the membrane have implications for either helping to preserve or disrupt the membrane potential that powers ATPase-mediated ATP production. Transcriptional responses to acid stress have been reported in the context of adaptation to wine-like conditions and include induction of classic chaperones such as *dnaK*, *grpE*, and *dnaJ* that facilitate protein conformational stability (11, 12). More specific responses to acid stress involve the induction of genes encoding alanine carboxypeptidase, which is involved in the maintenance of bacterial cell wall integrity, malate dehydrogenase/malate permease that contribute to cytoplasmic deacidification, and the gene *hsp18*, encoding the heat shock protein Lo18 (13, 14), a membrane-associated heat shock protein from the alpha crystallin family also known as gene *hsp20* (15).

Responses similar to those observed in O. oeni to acid stress have also been observed in response to ethanol stress. It is well known that ethanol can interfere with membrane structure, and in O. oeni, short-term fluidization is induced followed by membrane rigidification (16). The molecular chaperone Hsp20 is important in modulating this process (17–19). Furthermore, transcriptomic studies have demonstrated the dynamic and complex transcriptional changes induced by different ethanol concentrations, involving the differential expression of multiple molecular chaperones and genes associated with cell envelope biogenesis, MLF, and citrate metabolism (11, 12, 20).

The biochemical response of O. oeni to SO_2_ is less well studied, with the only reported toxic effect of SO_2_ being the inhibition of (F_1_F_o_) H^+^-ATPases; however, it is unclear whether this effect operates directly through an interaction with the ATPases or indirectly (e.g., impact on the cell membrane), as activity was measured through the consumption of ATP (5). A role for the molecular chaperone Hsp20 in the SO_2_ stress response was also proposed after a weak induction of this gene was observed under high SO_2_ conditions (21). Several mechanisms of action of SO_2_ have been hypothesized based on studies investigating other bacterial species, including damage to proteins, cell membranes, and DNA through nucleophilic substitutions and oxidative stress (22). However, the majority of these studies were conducted at pH values ranging from 5 to 7, which do not resemble the environment encountered by O. oeni in wine or grape juice containing SO_2_. Furthermore, high variability in the sensitivity of different bacterial species to SO_2_ has been demonstrated (23, 24).

This work describes an RNA sequencing (RNA-seq)-based investigation of the transcriptional changes induced in O. oeni strain AWRIB429 by SO_2_. Before SO_2_ treatments, continuous cultures of O. oeni were established in a semidefined medium (pH 3.5) to investigate the SO_2_-specific effects at wine-like pH. In contrast to previous transcriptomic studies performed in batch cultivations using this species, this approach enables the control of individual cultivation parameters and discrimination of transcriptional responses uniquely linked to SO_2_ exposure from changes associated with growth stage and cultivation parameters (25, 26). Our aim was to elucidate the protective mechanisms that O. oeni uses to counteract SO_2_ stress. A complete circularized reference genome was assembled from nanopore long-read sequence data to facilitate transcriptional analysis and improve gene-model annotations.

## RESULTS AND DISCUSSION

### Nanopore long-read genome assembly of Oenococcus oeni AWRIB429.

**FIG 1:**
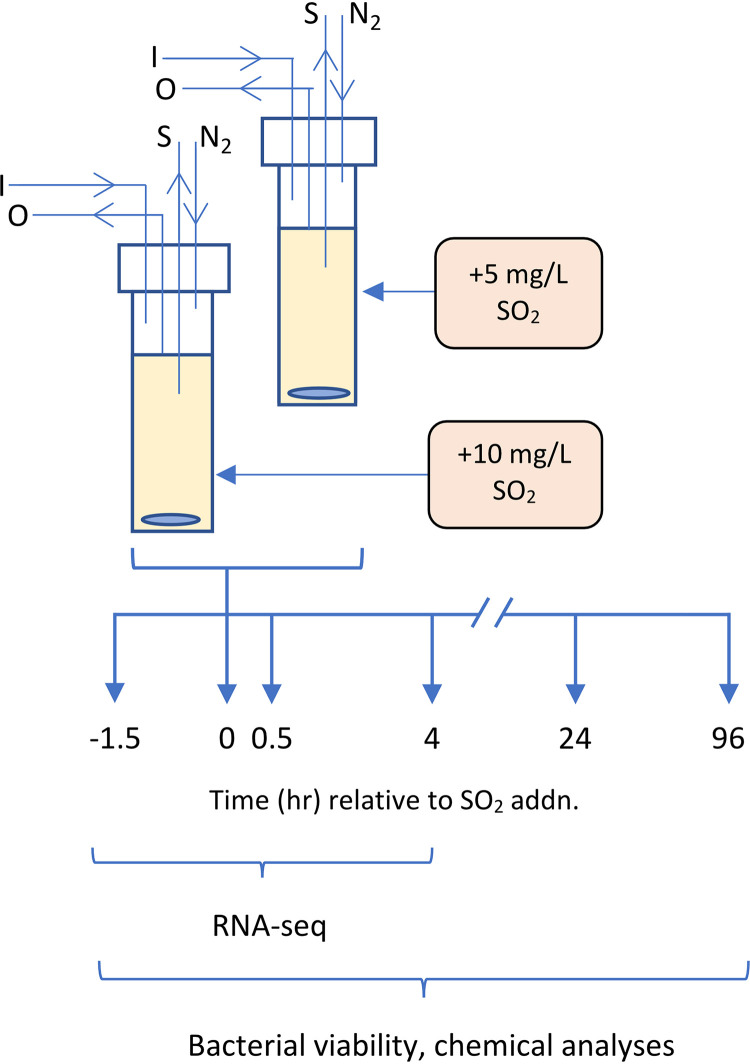
Schematic outline for investigation of O. oeni AWRIB429 response to SO_2_ stress during chemostat culture in semidefined medium (pH 3.5, 22°C, anaerobic). Cultures were dosed with 5 mg/liter and 10 mg/liter SO_2_ at 0 h, and samples were taken at indicated time points for RNA-seq analysis, determination of bacterial viability, and other chemical analyses. Chemostat cultures were performed in quadruplicate for each SO_2_ treatment. I, media inlet; O, effluent outlet; S, sample port; N_2_, nitrogen gas inlet; addn, addition.

**FIG 2:**
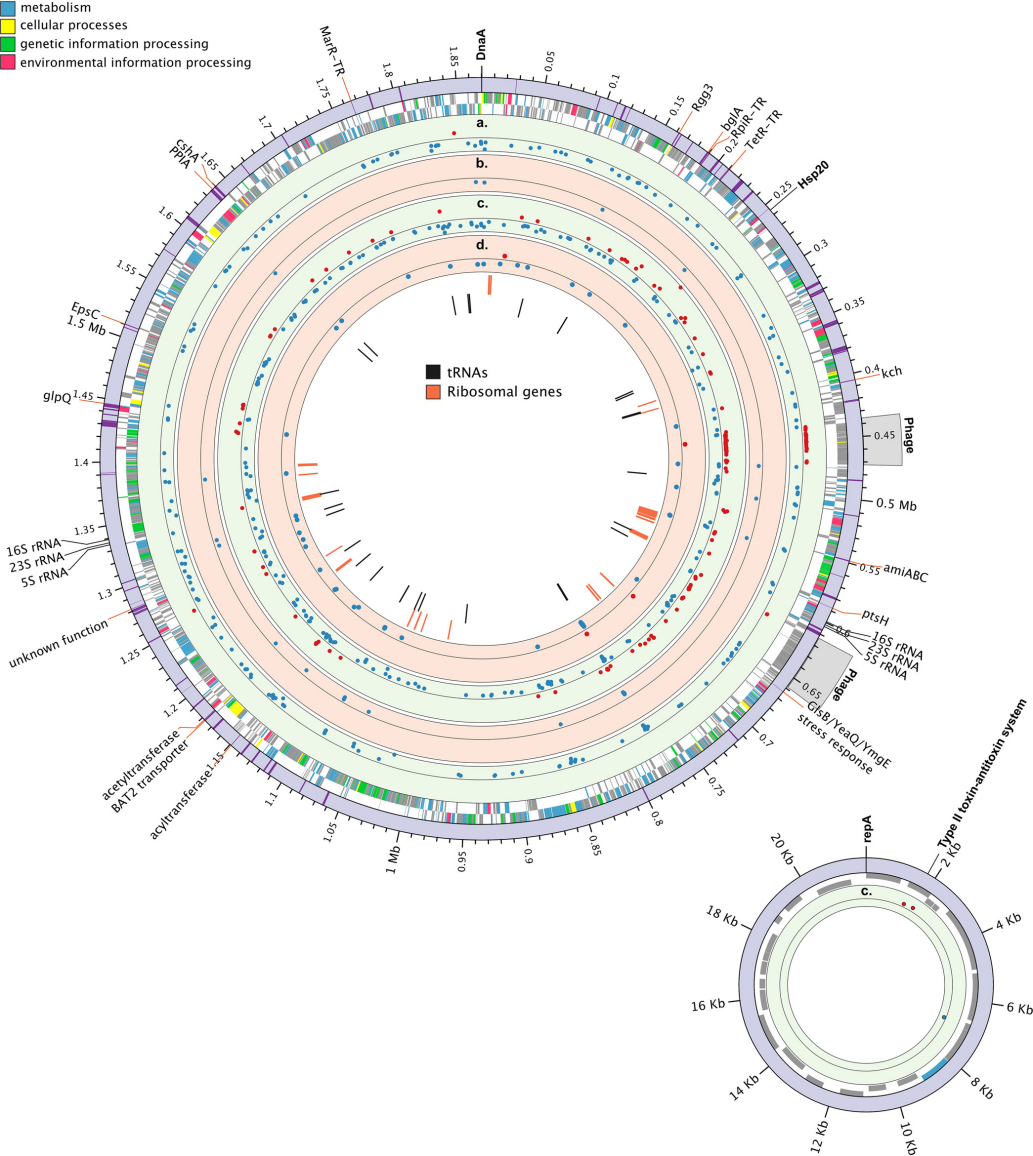
Complete circular representation of the chromosome and plasmid of Oenococcus oeni strain AWRIB429. From largest to smallest track: dark purple highlights within the outer purple track represent 99 wine stress-related genes reported by Margalef-Català et al. 2016 (11) and found in strain AWRIB429, with labeled genes observed as differentially expressed (1 < log_2_ FC < −1; adjusted *P* value of <0.005) in at least one of the SO_2_ treatments. Plus- and minus-strand ORFs are colored by KEGG functional categories as indicated in the outer legend. (a to d) Differentially expressed genes (up- and downregulated genes are represented as red and blue dots, respectively) observed under different SO_2_ treatments: 4 h (a) and 30 min (b) after addition of 10 mg/liter of SO_2_ and 4 h (c) and 30 min (d) after addition of 5 mg/liter of SO_2_. Top, middle, and bottom axes represent 7, 0, and −4 log_2_ FC in all tracks. The inner two tracks show the location of all ribosomal- and tRNA-coding ORFs.

The genome of O. oeni AWRIB429 was assembled using high-coverage nanopore long reads to obtain a contiguous assembly, improved gene-model annotations, and accurate transcriptome analysis derived from an experiment outlined in Figure 1. A complete circularized assembly was obtained for both the chromosome and plasmid present in this strain, with lengths of 1.89 Mbp and 21.9 kbp, respectively (Fig. 2). A total of 1,970 open reading frames (ORFs) were annotated, from which 1,237 were predicted to form part of multigenic operons, with a total of 380 operons and an average of 3.3 ORFs per operon. The longest predicted operons were composed of 16 and 14 ORFs, including a purine biosynthesis and ribosomal operon (14 ORFs) (Table S1 in the supplemental material). The largest predicted operon (16 ORFs) is not uniquely linked to one metabolic function and contains functionally unrelated genes (Table S1).

The fully contiguous assembly allowed the investigation of genomic elements such as tandem duplicated genes, temperate bacteriophages, and structural rearrangements between strain AWRIB429 and publicly available single contig genomes of O. oeni. Ten regions contained tandem gene duplications associated mainly with the acquisition of nutrients, including components of transporters for arabinose, arginine and hydroxymethylpyrimidine, an aspartate aminotransferase, and an aryl-6-phospho-beta-glucosidase. Two glycosyltransferases involved in cell wall synthesis and a block duplication comprising two genes, universal stress protein UspA and a putative Fe^2+^/Mn^2+^ transporter, were also observed as tandem duplicated (Table S2). Gene duplications generally occur as an evolutionary response to selective environmental pressure (27). That these genes are duplicated suggests that they may be relevant for the proliferation of this strain in the nutritionally deficient and stressful wine environment faced after alcoholic fermentation, where low concentrations of sugars such as arabinose and glycosides are available for growth (2) and amino acids such as arginine can confer increased tolerance to the acid environment (28).

Two complete temperate bacteriophages were identified within the genome of O. oeni with integrases belonging to previously defined groups int_A_ and int_D_ (Fig. 2) (29). The presence of a single incomplete bacteriophage with an int_D_ integrase was previously reported in strain AWRIB429 (30). This strain’s highly fragmented original assembly (59 contigs) was mapped back to the nanopore assembly to search for contigs mapping to both bacteriophage regions. The original assembly of strain AWRIB429 (31) did contain contigs that mapped to both predicted bacteriophages. However, correct assignment of these regions as bacteriophages was impaired due to the highly fragmented nature of the original assembly.

Interestingly, the predicted bacteriophage belonging to the int_D_ group showed high homology to, and contained all the structural components of, the previously sequenced O. oeni phage phi9805, which can excise, replicate, and release from O. oeni cells as well as confer potential superinfection immunity from other int_D_ phages (29). The second predicted bacteriophage shows a mosaic architecture with high levels of horizontal genetic exchange and modules showing homology to phages associated with multiple bacterial species (Table S3). A closer inspection of the chromosomal region associated with this predicted bacteriophage revealed a 2-fold increase in coverage when nanopore reads were mapped back to the genome of AWRIB429, with several reads clipped in the 5′ and 3′ ends of the bacteriophage region. This observation indicates that the predicted phage is entirely duplicated within the genome of strain AWRIB429 and that the long nanopore reads were not able to resolve this large duplication. Further research will be required to determine the lytic potential and stability of these O. oeni bacteriophages.

The presence of large genomic rearrangements within different O. oeni strains was investigated by comparing the genome of strain AWRIB429 against the publicly available *de novo* assembled single contig genomes belonging to diverse phylogenetic clades (Fig. S1). The high level of genetic variation within O. oeni strains has been well documented (30, 31). However, conservation of the overall genome organization has been reported with an absence of large structural genomic rearrangements (32). In agreement with Lorentzen et al. (32), no genomic rearrangements between strains UBOCC-A-315001 (clade D), CRBO_1381 (clade C), and the reference genome PSU-1 (clade A) were evident, despite belonging to phylogenetically distant clades. However, a comparison of these strains against strain AWRIB429 (clade A) revealed a large 670-kb chromosomal inversion flanked on both ends by rRNA operons (Fig. S1). Several long reads spanned the regions surrounding the inversion breakpoints, indicating that this inversion is not due to a misassembly and likely occurred through homologous recombination between the two rRNA operons that are present in an inverted orientation. To investigate if this chromosomal inversion is also present in O. oeni strains belonging to the previously reported phylogenetic clade B (32), two more strains corresponding to this clade (ATCC BAA-1163 and AWRIB787) were subjected to nanopore long-read whole-genome sequencing. Synteny analyses showed that only strain AWRIB787 contained the same large chromosomal inversion flanked by both rRNA operons (Fig. S1), suggesting that the inversion is likely widespread throughout O. oeni strains, without an apparent phylogenetic correlation. This asymmetrical inversion does not affect the origin of replication. However, it changes the length of the replichores and the distance between specific genes and the origin of replication. Variable distance between the origin and genetic elements has been suggested as a driver of selection in prokaryotes (33). The relevance of this structural rearrangement in the genome evolution of O. oeni will be elucidated once more long-read sequencing data from strains corresponding to different clades and isolation sources become available.

### Global transcriptional and physiological response to SO_2_.

The experimental approach used in this study (Fig. 1) was an important feature enabling a focused investigation of the transcriptional changes in O. oeni arising from SO_2_ stress. Foremost, continuous cultures of O. oeni strain AWRIB429 were established to accurately discriminate transcriptional responses linked to SO_2_ from other responses associated with growth stage and cultivation (25, 26). The composition of the continuous culture (CCOo) medium facilitated a satisfactory steady-state culture of O. oeni on a single carbon source (fructose) at wine-like pH (3.5). The two SO_2_ treatments used, low (5 mg/liter) and high (10 mg/liter), were determined from preliminary experiments investigating sublethal concentrations of SO_2_ in this system. Lastly, the transcriptomic comparisons were made at two time points, 30 min and 4 h, after SO_2_ addition (Fig. 1) to capture the peak adaptive transcriptional changes induced by SO_2_ stress in this bacterium species.

Differential gene expression analyses revealed major transcriptional differences between treatments (Fig. 2a to d.). A total of 406 genes were differentially expressed (DE) between all treatments and time points. Further, of the 99 genes in AWRIB429 that have previously been reported as DE (1 < log_2_ fold change [log_2_ FC] < −1) in strain PSU-1 in response to 12% (vol/vol) ethanol and pH 3.4 (11), only 17 were observed as DE after SO_2_ addition (Fig. 2). The limited number of common DE genes suggests a minor overlap between ethanol, pH, and SO_2_ stress responses. The limited overlap between DE genes could also be explained by differences in the experimental design presented in Margalef et al. (11).

Overall, it is noteworthy that the number of DE genes observed in the low-SO_2_ treatment (*N* = 363) was almost double those occurring in the high-SO_2_ treatment (*N* = 203). Specifically, in the low-SO_2_ treatment, transcriptional changes were readily observed after 30 min, with 4 and 43 genes observed as up- and downregulated, respectively (Fig. 2d). Examination of the specific function of the upregulated genes revealed that only one gene, corresponding to a glutaredoxin protein NrdH, was functionally annotated (Table 1). This gene is involved in the reduction of ribonucleotide reductases and contains a CXXC motif characteristic of dithiol glutaredoxins associated with oxidative protein damage (Table 1) (34, 35). Downregulated genes are predicted to be involved in several biological processes, including transcription regulation, oxidative stress, replication, cell wall assembly, and 12 aminoacyl-tRNAs (Table S4). In contrast, minor transcriptional changes were observed after 30 min in the high-SO_2_ treatment, with 27 downregulated genes, from which 12 were annotated as tRNAs and 1 as an acylphosphatase, and 8 genes were associated with transcription regulation, oxidative stress, replication, and respiration (Table S4).

**TABLE 1:** Differentially expressed genes between treatments grouped by functional category

		5 mg/liter SO_2_^*a*^^,^^*b*^		10 mg/liter SO_2_^*a*^^,^^*b*^	
Gene ID	Functional annotation	30 min	4 h	30 min	4 h
Protein and DNA damage					
J3U91_00272	Hsp20/alpha crystallin family protein: small heat shock protein	0.57	6.55	ND	0.68
J3U91_00467	nrdH; glutaredoxin	1.02	1.30	ND	1.17
J3U91_01815	trxA3; thioredoxin	ND	1.44	ND	ND
J3U91_01735	trxA2; thioredoxin	−1.44	−1.16	−1.02	−1.88
J3U91_00500	msrA; peptide-methionine (*S*)-*S*-oxide reductase	−0.31	−1.08	ND	−0.43
J3U91_00562	clpL; ATP-dependent Clp protease ATP-binding subunit ClpL	ND	2.44	ND	0.62
J3U91_00560	clpP; ATP-dependent Clp protease, protease subunit	ND	1.86	ND	0.70
J3U91_00629	clpE; ATP-dependent Clp protease ATP-binding subunit	ND	1.68	ND	0.51
J3U91_00478	clpP; ATP-dependent Clp protease, protease subunit	0.65	1.24	ND	0.99
J3U91_00327	uvrB; excinuclease ABC subunit B	ND	1.19	ND	ND
J3U91_01004	recU; recombination protein U	−0.51	−1.30	ND	−1.31
J3U91_00004	recF; DNA replication and repair protein	−0.81	−0.93	−0.76	−1.42
Carbohydrate metabolism, nutrient uptake, and energy					
J3U91_00050	Diacetyl reductase	ND	1.23	ND	0.37
J3U91_00122	rbsK; ribokinase	−0.45	−1.08	ND	−0.41
J3U91_00136	rpiA; ribose 5-phosphate isomerase A	−0.70	0.78	−0.60	−1.18
J3U91_00145	acyP; acylphosphatase	ND	1.23	−1.22	−1.90
J3U91_00197	l-Threonine 3-dehydrogenase	ND	1.03	ND	0.56
J3U91_00206	6-Phospho-beta-glucosidase	ND	1.26	ND	ND
J3U91_00218	gatB; galactitol PTS system EIIB component	0.69	1.29	ND	0.59
J3U91_00228	mntH; manganese transport protein	ND	−1.08	ND	−0.55
J3U91_00250	HIBADH; 3-hydroxyisobutyrate dehydrogenase	ND	1.12	ND	ND
J3U91_00265	celB; cellobiose PTS system EIIC component	−0.56	−1.51	−0.66	−1.71
J3U91_00331	uraA; uracil permease	ND	−1.73	ND	−0.60
J3U91_00348	SORD; l-iditol 2-dehydrogenase	ND	1.18	ND	ND
J3U91_00375	citD; citrate lyase subunit gamma (acyl carrier protein)	ND	1.02	ND	0.72
J3U91_00376	citE; citrate (pro-3*S*)-lyase subunit beta	ND	0.99	ND	0.63
J3U91_00377	citF; citrate lyase subunit alpha/citrate CoA-transferase	ND	1.09	ND	0.71
J3U91_00415	kch; voltage-gated potassium channel	−0.57	−1.12	ND	−1.21
J3U91_00739	adhP; alcohol dehydrogenase, propanol-preferring	ND	1.42	ND	ND
J3U91_00916	APA; basic amino acid/polyamine antiporter, APA family	−0.60	−1.52	−0.36	−0.88
J3U91_00927	pgl; 6-phosphogluconolactonase	ND	1.26	ND	0.61
J3U91_00980	arcA; arginine deiminase	−0.38	−1.61	ND	−0.71
J3U91_01368	lysY; putative lysine transport system ATP-binding protein	0.60	1.10	ND	0.71
J3U91_01401	speG; diamine *N*-acetyltransferase	ND	−1.11	ND	−0.68
J3U91_00728	PTS glucose transporter subunit IIA	ND	ND	ND	−1.12
J3U91_01460	ATPF0A; F-type H^+^-transporting ATPase subunit a	ND	−0.94	ND	−1.13
J3U91_01562	Glycerophosphoryl diester phosphodiesterase	0.72	1.16	0.66	0.81
J3U91_01579	GntP; gluconate:H^+^ symporter	ND	1.07	ND	ND
J3U91_01581	ulaA; ascorbate PTS system EIIC component	0.64	1.12	0.66	0.67
J3U91_01583	ulaC; ascorbate PTS system EIIA or EIIAB component	ND	1.10	ND	ND
J3U91_01672	mleP; malate permease	0.52	1.14	0.37	0.91
J3U91_01673	mleA; malolactic enzyme	0.45	1.15	0.31	0.84
J3U91_01682	abfA; alpha-l-arabinofuranosidase	ND	1.05	ND	0.61
J3U91_01734	APA; basic amino acid/polyamine antiporter, APA family	ND	−1.34	ND	−0.90
J3U91_01750	alr; alanine racemase	−0.29	−1.25	−0.29	−0.75
J3U91_01778	kdgR; LacI family transcriptional regulator, kdg operon repressor	ND	1.29	ND	ND
J3U91_01817	alsD; acetolactate decarboxylase	ND	0.57	ND	0.40
J3U91_01828	glcU; glucose uptake protein	ND	−1.45	ND	−0.65
J3U91_01978	celC; cellobiose PTS system EIIA component	ND	−1.24	ND	ND
J3U91_01979	celA; cellobiose PTS system EIIB component	ND	−1.78	ND	−1.63
Cell envelope and division					
J3U91_00162	divIC; cell division protein	−0.69	−0.98	−0.48	−1.26
J3U91_00301	pgmB; beta-phosphoglucomutase	ND	2.02	ND	ND
J3U91_00444	polysaccharide biosynthesis protein	−0.64	−1.10	−0.62	ND
J3U91_00577	amiABC; *N*-acetylmuramoyl-l-alanine amidase	−0.46	−1.06	ND	ND
J3U91_00641	ltaS; lipoteichoic acid synthase	−0.51	−1.21	−0.53	−1.10
J3U91_00932	division/cell wall cluster transcriptional repressor MraZ	ND	−1.74	ND	−1.63
J3U91_00940	ftsA; cell division protein	ND	−1.02	ND	ND
J3U91_00944	Cell division protein	−0.60	−2.04	−0.39	−1.10
J3U91_01123	dgkA; undecaprenol kinase	−0.72	−0.53	−0.65	−1.12
J3U91_01229	FemA; peptidoglycan bridge formation glycyltransferase	ND	1.35	ND	ND
J3U91_01307	cwlO; peptidoglycan dl-endopeptidase	ND	−1.12	ND	−0.72
J3U91_01479	clsA_B; cardiolipin synthase A/B	−0.39	−1.38	ND	−1.00
J3U91_01480	mreD rod shape-determining protein	−0.82	−2.32	−0.83	−2.49
J3U91_01481	mreC; rod shape-determining protein	−0.52	−1.21	−0.44	−0.75
J3U91_01533	LysM peptidoglycan-binding domain-containing protein	ND	−1.41	ND	ND
J3U91_01550	rfbB; dTDP-glucose 4,6-dehydratase	ND	1.24	ND	0.71
J3U91_01551	rfbC; dTDP-4-dehydrorhamnose 3,5-epimerase	ND	1.55	ND	0.72
J3U91_01552	rfbA; glucose-1-phosphate thymidylyltransferase	ND	1.92	ND	ND
J3U91_01614	Glycosyltransferase eps cluster 2	ND	−1.31	ND	ND
J3U91_01615	Putative glycosyltransferase eps cluster 2	ND	−1.14	ND	ND
J3U91_01619	Putative glycosyltransferase eps cluster 2	−0.42	−1.13	ND	ND
J3U91_01625	Capsular polysaccharide biosynthesis protein	ND	−1.68	ND	ND
J3U91_01854	Glycosyltransferase eps cluster 1	ND	−1.07	ND	ND
J3U91_01858	Putative glycosyltransferase eps cluster 1	−0.31	−1.01	ND	ND
J3U91_01902	murA; UDP-*N*-acetylglucosamine 1-carboxyvinyltransferase	−0.90	−1.45	−0.63	−1.02
J3U91_01940	bacA; undecaprenyl-diphosphatase	ND	−1.73	ND	−0.86
J3U91_01941	tagU; peptidoglycan teichoic acid transferase	−1.56	−3.37	−0.98	−2.71

a Changes in gene expression are represented as log_2_ fold change (log_2_ FC) between time point and control condition before SO_2_ addition.

b Shading shows genes with a log_2_ FC of 1 < log_2_ FC < −1, and treatments in which gene expression changes show an adjusted *P* value of >0.005 are represented as not detected (ND).

A genome-wide transcriptional remodeling was observed after 4 h of the low-SO_2_ treatment, with 139 and 215 genes up- and downregulated, respectively (Fig. 2c). The highest induced and repressed genes, with a log_2_ FC of 6.55 and −3.73, correspond to the small heat shock protein Hsp20 and a LysR-type transcriptional regulator (Table S4). Hsp20 (referred to as Hsp18 in several studies) has previously been identified as a response to ethanol and acid stress in O. oeni (22) and has also been associated with the SO_2_ stress response (36). The involvement of this protein in SO_2_ stress is discussed in subsequent sections.

Three genes located in the assembled plasmid were also DE in the low-SO_2_ treatment. Two of these plasmid-associated genes relating to a putative type II toxin/antitoxin system were upregulated (Fig. 2). The physiological role of type II toxin/antitoxin systems has not been investigated in O. oeni specifically. However, in Escherichia coli several functions, such as stabilization of mobile elements, abrogation of bacteriophage infections, and antibiotic tolerance, have been attributed to specific toxin/antitoxin systems (37).

Compared to the low-SO_2_ treatment, considerably fewer transcriptional changes were observed in the high-SO_2_ treatment after 4 h (Fig. 2a). In high SO_2,_ most of the DE genes were downregulated (*N* = 30 and *N* = 173 genes up- and downregulated, respectively). The most highly induced genes included a tRNA, an IS5 family transposase, and the glutaredoxin protein NrdH (Table S4). The highest transcriptional repression was observed in gene *ybcJ*, encoding a ribosome-associated protein (Table S4). Interestingly, a large region encompassing 39 contiguous genes that form part of one of the two predicted complete temperate bacteriophages was upregulated (*N* = 23 genes with a log_2_ FC of >1) in both the high- and low-SO_2_ treatments after 4 h (Fig. 2). This region also contains an accessory locus encoding the glutaredoxin protein NrdH, which was upregulated after 30 min and 4 h of low-SO_2_ addition (Table 1), possibly providing a beneficial function in the O. oeni SO_2_ stress response. Whether the temperate bacteriophage is induced under SO_2_ stress, as observed in phages of other bacterial species under stressful conditions (38), will require further investigation.

**FIG 3:**
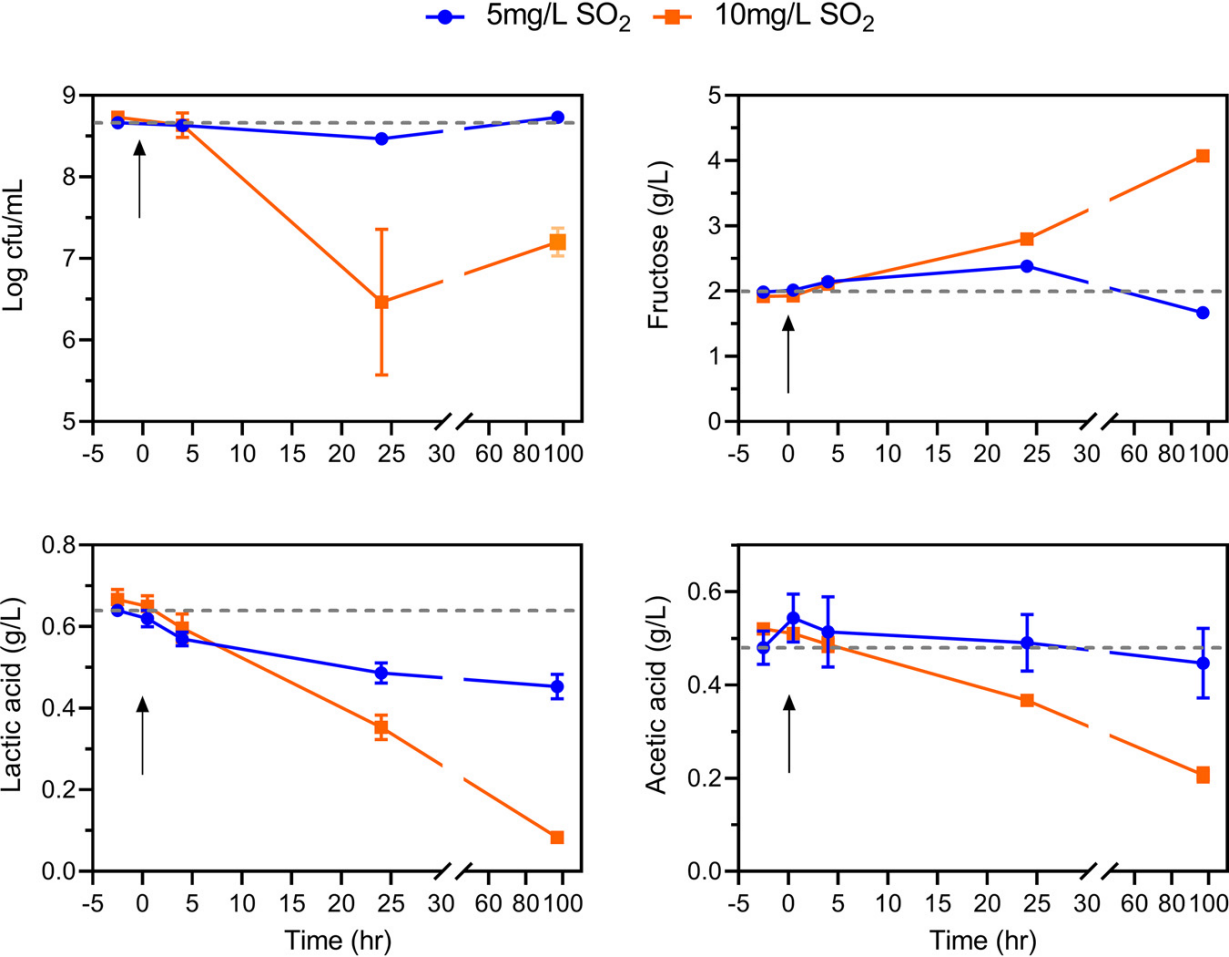
Effect of SO_2_ exposure on viable cells and metabolism of fructose and lactic and acetic acids by Oenococcus oeni AWRIB429 during continuous culture. Arrows represent the time points of SO_2_ addition. Data points represent the average of three replicate cultures and standard deviations. Dotted lines outline initial measurements before SO_2_ in the low-SO_2_ treatment (5 mg/liter SO_2_).

Cell viability, fructose consumption, and lactic and acetic acid production were monitored throughout the experiment (Fig. 3). In concert with transcriptional changes, slight modulations in the concentrations of lactic and acetic acids and fructose were observed after 4 h in both SO_2_ treatments, although cell viability remained relatively constant over this time (Fig. 2). However, greater latent impacts of the SO_2_ treatments on these parameters occurred after extended culture (24 and 96 h), particularly in the high-SO_2_ treatment (Fig. 3). In this case, cell viability decreased over 100-fold to 6.5 ± 0.9 log CFU/ml after 24 h and showed slight recovery to 7.2 ± 0.1 log CFU/ml after 96 h. Over the same period, the concentration of fructose increased to 4.1 g/liter after 96 h and that of lactic and acetic acids concomitantly decreased to 0.1 and 0.2 g/liter, respectively. In contrast, the latent impacts of the low-SO_2_ treatment were comparatively minimal, with cell viability and the concentrations of fructose and acetic acid remaining at pre-SO_2_ treatment concentrations after 24 and 96 h (Fig. 3).

The global transcriptional changes and growth kinetics observed in the low-SO_2_ treatment suggest that this SO_2_ concentration was sufficient to induce a transcriptional stress response in O. oeni that allowed survival and growth maintenance. In contrast, the data suggest that cells exposed to the high-SO_2_ treatment could not rapidly remodel their transcriptome to counteract the long-term damage induced by SO_2_. As a result, the viable cell population decreased substantially after 24 h due to a combination of cell death and dilution (Fig. 3).

### SO_2_ induces protein damage and recycling mechanisms.

The toxic mechanisms of SO_2_ toward O. oeni are currently unknown; however, modes of action have been suggested, including adduct formation via nucleophilic attack (39) or, more likely in winemaking environments, oxidative damage through autoxidation of SO_2_ and generation of sulfuroxy radicals (22). Under oxidative stress caused by radicals, proteins containing cysteine and methionine residues are prone to oxidation at their electron-rich sulfur atoms. Bacteria can counteract oxidative protein damage by reducing cysteine and methionine residues to their thiol state with the help of oxidoreductases, including thioredoxins, glutaredoxins, and methionine sulfoxide reductases (40). The thioredoxin system has been recently characterized in O. oeni (41) and shown to be upregulated under oxidative and heat shock stress (36). Interestingly in the current study, genes associated with the repair of oxidized proteins and oxidative stress were upregulated in the short- and long-term response (Table 1; Table S4). These included genes encoding the glutaredoxin protein NrdH, the thioredoxin TrxA and two other oxidoreductases previously shown to be involved in the oxidative stress response (42, 43), an NADH-dependent flavin oxidoreductase and an NADPH-dependent quinone oxidoreductase (Table S4). While the upregulation of these genes does suggest oxidative stress reactions, such as oxidation of cysteine residues, the mechanism of sulfite radical formation from SO_2_ under these conditions is unclear. Nevertheless, some mechanisms for sulfite radical formation have been previously proposed, including autoxidation through catalysis with transition metals such as manganese (44) and enzymatic oxidation with peroxidases (45). Furthermore, it is also possible that protein damage occurs through both oxidative damage and nucleophilic substitution. While oxidoreductases, such as thioredoxins and glutaredoxins, will aid in repairing proteins, upregulation of the Clp machinery as observed here, including the ATP-dependent ClpP protease subunit and two ATP-binding subunits ClpL and ClpE (Table 1), indicates increased protein turnover and suggests that SO_2_ also irreversibly damages proteins. Overall, the data are consistent with intracellular protein damage being one of the main mechanisms of SO_2_ toxicity in O. oeni.

### *Hsp20* is upregulated in response to SO_2_ exposure.

Molecular chaperones play an essential role in the response of LAB against environmental stresses by protecting and refolding proteins, allowing them to continue functioning during exposure to stress (46). In O. oeni, a small heat shock protein belonging to the Hsp20 family is considered the most important molecular chaperone associated with the response to heat, ethanol, acid, and oxidative stress (15, 22). Besides its demonstrated role in protein protection (19, 47), studies have shown its involvement in the rigidification of the membrane lipid bilayer after stress-induced fluidization (13, 19). Aside from Hsp20, other molecular chaperones, including DnaJ, DnaK, GrpE, and GroESL, have been associated with the general wine stress response (without SO_2_) (11, 12); however, their specific mechanisms of action have not been investigated in O. oeni. From the molecular chaperones observed in these previous studies, only *hsp20* expression was upregulated after 4 h of exposure to the low-SO_2_ treatment (Fig. 2; Table 1). *Hsp20* had the highest fold change increase (log_2_ FC of 6.55) across all treatments and time points. This result is consistent with the study of Guzzo et al. (21) in which an induction in *hsp20* expression was observed after the addition of SO_2_, albeit at much higher concentrations than were used here (60 mg/liter). The sole upregulation of *hsp20* among all the reported chaperones confirms its importance in the response of O. oeni toward SO_2_ and may be essential to stabilize, and prevent the aggregation of, damaged proteins.

### DNA damage.

Autoxidation and the formation of sulfuroxy radicals as well as the nucleophilic nature of SO_2_ have been associated with DNA damage through cleavage of double-stranded DNA (44, 48), mutations (49–51), and hydroxylation of guanosine (52). Bacteria possess several DNA repair mechanisms to counteract DNA damage (53); however, a recent report suggests that O. oeni lacks a functional mismatch repair mechanism (54). Without mismatch repair, O. oeni would rely on other excision repair mechanisms to counteract DNA damage induced by SO_2_. Three genes that form part of the recombinational and nucleotide excision repair systems were DE after 4 h of SO_2_ addition (Table 1). Of these, the only upregulated gene was *uvrB*, encoding the excinuclease ABC subunit B of the excision repair system (Table 1). Interestingly, *uvrB* activity, and therefore excision repair, has been demonstrated as essential to counteract the mutagenic effects of SO_2_ in Salmonella enterica serovar Typhimurium (50, 55). *UvrB* plays an important role in locating damaged DNA before incision (56), and its upregulation is consistent with increased DNA damage in cells exposed to SO_2_. It has been reported that the mutagenic effects through nucleophilic addition require high concentrations of SO_2_ (1 M, 1,040 mg/liter), while at lower concentrations, such as those common in wine, oxidative damage through bisulfite-generated free radicals is more likely to be responsible for mutagenic DNA damage (50).

### Cell envelope.

The effects of SO_2_ on the cell envelope of O. oeni are currently unknown. Based on the observed impact on the activity of (F_1_F_o_) H^+^-ATPases, it has been hypothesized that SO_2_ could interact with the cell membrane (5). Disruption of cell structure and cytoplasmic disordering has also been reported in O. oeni cells exposed to high SO_2_ concentrations (23). Multiple genes associated with cell envelope biosynthesis were downregulated after SO_2_ addition (Table 1), of which *tagU*, encoding a peptidoglycan teichoic acid transferase, and *mreD*, a rod shape-determining protein, were among the most downregulated genes in both the low- and high-SO_2_ treatments (Table 1). The genes *pgmB* and *rfbABC* involved in the synthesis of dTDP-l-rhamnose and *femA*, encoding a peptidoglycan bridge formation protein, were upregulated after 4 h of low SO_2_ exposure (Table 1). The transcriptional changes are consistent with the interaction of SO_2_ with the cell envelope of O. oeni. The observed DE genes indicate a remodeling of the cell wall through a downregulation of genes associated with the synthesis of teichoic and lipoteichoic acids (Table 1) and an increase of interpeptide peptidoglycan bridges. Further, dTDP-l-rhamnose is an important precursor for the synthesis of exopolysaccharides (EPS) in O. oeni (57–59), which can have a protective role under stressful wine conditions (60). The upregulation of genes associated with the synthesis of dTDP-l-rhamnose are consistent with an increased requirement for this monomer to support the synthesis of heteropolysaccharides. However, none of the genes associated with the EPS cluster were upregulated. Nevertheless, the results suggest that EPS may serve a role in the stress response of O. oeni against SO_2_ stress and should be further investigated.

### Metabolic adaptations to SO_2_.

Lactic acid bacteria can adapt to environmental stress by modulating the flux through alternative pathways for sugar consumption and making use of diverse carbon sources (46). It is well known that O. oeni relies on pathways, such as MLF and citrate metabolism, to counteract common wine stresses such as low pH and ethanol (22). However, the adaptations in relation to SO_2_ stress are less well understood, with a single study reporting a reduction in the activity of (F_1_F_o_) H^+^-ATPases (5).

The kinetics associated with the consumption of fructose and DE genes associated with carbohydrate metabolism were examined to gain further insight into the metabolic adaptations of O. oeni to SO_2_. In the low-SO_2_ treatment, a minor reduction in fructose consumption occurred over 24 h, accompanied by an early, short-duration spike in acetic acid production after 30 min (Fig. 3). Despite the recovery in fructose consumption after 24 h, lactic acid production continued to decline, indicating that heterofermentative flux was redirected toward the production of alternative carbon compounds. The transcriptomic data for this treatment showed an upregulation of several genes associated with carbohydrate metabolism, including genes involved in the consumption of citric and malic acid, production of diacetyl and acetic acid, and two glycosidases (Table 1). Two phosphotransferase system (PTS) transporters (ascorbate and galactitol), an H^+^:gluconate symporter, and an amino acid ABC transporter were also upregulated (Table 1).

Taken together, the data are consistent with a remodeling of carbon metabolism during mild SO_2_ stress (low-SO_2_ treatment). Even though malic acid was not a component of the CCOo medium, the upregulation of genes related to MLF metabolism highlights the importance of this pathway in the response of O. oeni to SO_2_. A relationship between the MLF operon’s transcription and ATPase activity has previously been reported (61), in which case the induction of MLF-related genes may indicate that ATPase activity is not inhibited at the concentrations of SO_2_ used in this study.

The physiological and transcriptomic changes in carbohydrate metabolism in the high-SO_2_ treatment contrasted substantially to those of the low-SO_2_ treatment. Under high-SO_2_ conditions, a much greater and consistent reduction in fructose consumption and lactic and acetic acid production occurred over 96 h (Fig. 3). These changes in consumption and production were reflected in the downregulation of several genes associated with carbohydrate metabolism, including a ribose 5-phosphate isomerase, PTS glucose transporter, and (F_1_F_o_) H^+^-ATPases (Table 1). Furthermore, the comparative lack of DE genes associated with carbohydrate metabolism as observed in the low-SO_2_ treatment suggests that redirection of metabolic flux toward alternative carbon compounds did not occur.

## CONCLUSION

This study investigated the transcriptional changes linked to SO_2_ stress in the wine bacterium O. oeni and provides evidence for how this compound interacts with the cellular components of this species. A variety of transcriptional changes were observed relating to protein and DNA damage, carbohydrate metabolism, and energy production as well as the cell envelope and cell division. Notably, the profile of DE genes is consistent with a model of SO_2_ action in which SO_2_ reacts with intracellular proteins causing irreparable damage, likely through oxidative mechanisms. The importance of the molecular chaperone Hsp20 in response to SO_2_ by this species has also been confirmed, making this gene a potential target for the development of SO_2_-tolerant strains. Upregulation of genes involved in MLF in the absence of l-malic acid was observed, providing clear evidence of the vital role that metabolism of malic acid plays as part of a multifaceted response of O. oeni to SO_2_ stress. Upregulation of genes involved in citrate and diacetyl metabolism also points toward a role for these systems in response to SO_2_ stress and should be further investigated. The first nanopore-based complete genome assemblies for O. oeni are also reported along with the DNA sequence of two bacteriophages in strain AWRIB429.

## MATERIALS AND METHODS

### Media and continuous culture.

*Bacteria strain and cell preculture.* Oenococcus oeni strain AWRIB429 was used for the RNA-seq experiments. This strain is an isolate from the commercial malolactic starter culture preparation Lalvin VP-41 (Lallemand). Its genomic sequence has been reported by Borneman et al. (31). Two O. oeni strains belonging to the previously reported phylogenetic clade B (32) were also used for whole-genome sequencing and comparative genome analyses with published genomes of strains belonging to clades A, C, and D.

Before experimental usage, bacterial cells of strain AWRIB429 were passaged through three consecutive preculture stages, each at 27°C in a nitrogen atmosphere. Cells from cryogenic (−80°C) storage were initially grown for 7 days on a modified de Man, Rogosa and Sharpe agar medium (MRSFM) (MRS [Oxoid, Australia] supplemented with d-fructose [10 g/liter] and dl-malic acid [6 g/liter], pH 5.0), from which colonies were subsequently cultured in MRSFM liquid medium (8 ml) for an additional 6 days. Cells from the latter culture were then inoculated (2% [vol/vol]) into a final starter culture medium prepared from that used for continuous culture of O. oeni (CCOo pH 4.0, 50 ml) described below and cultured for 9 days.

*Medium for continuous culture of* O. oeni. An important requirement for this study was implementing a culture medium with low SO_2_-binding capacity and that supported a high biomass concentration during continuous culture at wine pH (pH 3.5). To this end, a culture medium based on that described by Wells and Osborne (62) and Hood (63) was used with modifications, including replacement of yeast extract with bacteriological peptone to avoid precipitation at low pH (indicated by Margalef-Català et al. [11]), supplementation with vitamins (described in Schmidt et al. [64]) from a 1,000× stock solution, and omission of ferric chloride, calcium chloride, and malic acid. d-Fructose was the sole carbon source. Preliminary continuous culture experiments with CCOo medium revealed that 5 g/liter d-fructose was optimal for bacterial growth, and the pH of the medium remained constant. The CCOo medium was composed of bacteriological peptone (Amyl) (20 g/liter), Casamino Acids (Difco) (5 g/liter), d-fructose (5 g/liter), potassium hydrogen tartrate (2.5 g/liter), K_2_HPO_4_ (2.0 g/liter), MgSO_4_·7H_2_O (1.0 g/liter), MnSO_4_·H_2_O (0.02 g/liter), Tween 80 (1 ml/liter), and vitamins stock solution (1 ml/liter), adjusted to pH 3.5 with HCl. All liquid media were sterilized by filtration through a 0.2-μm-pore-size membrane.

*Continuous culture.* A continuous culture was used to facilitate the study of the transcriptional response of O. oeni cells to SO_2_, without extrinsic interferences arising from batch culture. Anoxic conditions were used to avoid the reaction of SO_2_ with oxygen. Eight replicate chemostat cultures of exponential-phase O. oeni AWRIB429 cells were prepared using small-scale (250-ml) bioreactors. Each bioreactor was fitted with an N_2_ gas inlet (0.2-μm-membrane filtered, flowrate of 2.5 cm^3^/min) to maintain anaerobiosis and slight positive pressure in the headspace to drive effluent outflow and ports for media inflow and sample collection. The CCOo medium was supplied to bioreactors via a peristaltic pump (average flowrate of 7.6 ± 0.3 ml/h; dilution rate of 0.030 ± 0.001 h^−1^). Bioreactors were incubated at 22°C and constantly stirred (300 rpm). Before experimentation, continuous cultures were equilibrated for 10 days (7.2 culture volumes). At the commencement of experimentation, the average viable O. oeni population of the 8 bioreactors was 5.0 ± 0.8 × 10^8^ CFU/ml.

### SO_2_ treatments and sampling.

A schematic outline of the continuous culture apparatus and experimental approach is shown in Fig. 1. Before addition of SO_2_, aqueous stock (100× volume) solutions for 5 mg/liter and 10 mg/liter SO_2_ additions were separately prepared from potassium metabisulfite, the concentrations of which were 1.1-fold greater than required to compensate for SO_2_ loss during preparation and following addition to bioreactors. The addition rates were confirmed through measurement of free and total SO_2_ of the stock solutions. These were 4 and 9 mg/liter of total SO_2_ (all of which was unbound in the stock solution) for the 5 and 10 mg/liter treatments, respectively. Each SO_2_ treatment was added to three bioreactors (0-h time point). Samples (2 ml) were aseptically taken from each bioreactor for RNA-seq, cell viability, and high-performance liquid chromatography (HPLC) analyses (Fig. 1). Samples for RNA-seq analyses were taken before and 30 min and 4 h after SO_2_ addition (Fig. 1). These time points were selected based on previous transcriptomic and proteomic studies reporting induction of the adaptive transcriptional response within the first minutes to hours after exposure to environmental stress in yeast and lactic acid bacteria (65–68). Samples were initially centrifuged (approximately 30,000 × *g*, 4°C, 1.5 min). Cell pellets were snap-frozen in liquid nitrogen and stored at −80°C for subsequent RNA-seq, and supernatants were stored separately at −20°C for HPLC analyses. The time from removal of the sample from the bioreactor to freezing was less than 3 min. An additional sample (noncentrifuged) was used for the determination of bacterial viability.

### Analytical methods.

The determination of free and total SO_2_ was undertaken by the Australian Wine Research Institute Commercial Services laboratory using a discrete analyzer (Thermo Gallery). Reagents and absorbance wavelengths for the determination of free and total SO_2_ in this method were pararosaniline and formaldehyde (575 nm) and 5,5′-dithio-bis-2-nitrobenzoic acid (412 nm), respectively. The concentrations of lactic and acetic acids and fructose were determined by HPLC using a Bio-Rad HPX-87H column as described by Nissen et al. (69). The concentration of viable O. oeni cells was determined by spot plating duplicate 25-μl aliquots of serially diluted (0.1% [wt/vol] bacteriological peptone [Amyl Media]) samples onto MRSFM agar. The agar plates were incubated at 27°C in an N_2_ atmosphere for 8 to 12 days, and resultant bacterial colonies were enumerated.

### DNA extraction.

For DNA extraction, O. oeni strains AWRIB429, AWRIB787, and ATCC BAA-1163 were grown to an optical density at 600 nm (OD_600_) of 1 in MRS medium (Amyl Media) supplemented with 20% apple juice. Cells were pelleted by centrifugation (15 min, 3,000 × *g*) and washed in 1 ml of GTE buffer (50 mM glucose, 25 mM Tris pH 8, and 10 mM EDTA). For cell lysis, pellets were resuspended in 1 ml of GTE buffer containing 20 mg/ml lysozyme and incubated for 3 h at 37°C. One hundred microliters of 10% SDS was then added and mixed before incubation for 40 min at 37°C. Then, 2 μl of RNase A (40 μg/μl) (Qiagen, Australia) was added, vortexed, and incubated for 20 min at 37°C. Twenty microliters of proteinase K (20 μg/μL) (New England BioLabs, Australia) was then added, mixed, and incubated overnight at 37°C. Finally, DNA was isolated and purified using a GeneElute bacterial genomic DNA kit (Sigma, Australia) following the manufacturer’s instructions.

### Long-read sequencing, genome assembly, and annotation.

Libraries for nanopore sequencing were prepared using the SQK-LSK109 ligation kit and loaded into a FLO-MIN106 R9 flow cell. Fast5 files were base called, demultiplexed, and adapter trimmed using Guppy v3.2.1 (Oxford Nanopore Technologies, Oxford, UK) with the high-accuracy model and a minimum quality score of 7, obtaining a final approximate coverage of 245× per strain.

The genomes of O. oeni strains AWRIB429, AWRIB787, and ATCC BAA-1163 were assembled and circularized using Unicycler v.0.4.8 (70) and then polished with long reads using Racon v.1.4.13. A final polish was performed in the genome sequence of strain AWRIB429 with Pilon v.1.23 (71) using 2 × 150-bp synthetic Illumina reads obtained from the genome assembly of this strain available in NCBI (assembly accession GCA_00017535). Gene and functional annotations were performed with PROKKA v.1.14.16 (72), including the gene models and functional annotations of O. oeni PSU-1 (assembly accession GCF_000014385). Further functional protein annotations were performed with KEGG (73) and InterProScan 5 (74). The prediction of temperate bacteriophages was performed using PHASTER (75).

### RNA isolation and sequencing.

Cell pellets from three bioreactors per treatment and time point (0 min, 30 min, and 4 h) were thawed on ice and mixed with 350 μl of lysis buffer (LB buffer from a PureLink RNA mini kit manual with 1% 2-mercaptoethanol) and 200 mg of 0.1-mm acid-washed glass beads (Sigma, Australia) in 1-ml screw-cap tubes. Cells were lysed in a Precellys bead beater (Bertin Technologies, France) (8,000 rpm, 15 s × 3) and placed on ice. Supernatant (350 μl) was then extracted and mixed with an equal volume of 70% RNase-free ethanol (Life Technologies, USA). RNA was then extracted and purified using a PureLink RNA minikit (Life Technologies, USA) following the manufacturer’s instructions. Final samples were DNase treated (DNase I, New England BioLabs, Australia) following the manufacturer’s instructions.

Samples were sent for rRNA depletion, library preparation, and sequencing to the Ramaciotti Centre for Genomics (University of New South Wales, Sydney, Australia). Sequencing libraries were prepared using a Zymo-Seq RiboFree total RNA library kit (Zymo Research, USA) and sequenced in an Illumina NextSeq 500 using a high-output flow cell and 1 × 75-bp chemistry.

### Differential gene expression analysis.

Illumina single-end reads were quality trimmed using Trimmomatic v.0.38 (76). Creation of a genome index and mapping of the Illumina reads to the genome of O. oeni AWRIB429 was performed using STAR v.2.7.3a (77). Counting of reads mapping to each genomic feature was performed using featureCounts v.2.0.0 (78). Read count tables were imported into R (79), and features with 0 counts in all samples were removed. Differential gene expression analyses were performed using the DESeq2 package v.1.24.0 (80) with default parameters (sample-wise size factor normalization, Cox-Reid dispersion estimate, and the Wald test for differential expression), comparing each time point after SO_2_ (0.5 and 4 h) against the corresponding time point of −1.5 h since SO_2_ addition (Fig. 1). Features with a log_2_ FC of 1 < log_2_ FC < −1 and an adjusted *P* value of <0.005 were considered for further analysis.

### Data availability.

The genome sequences of strains AWRIB429, ATCC BAA-1163, and AWRIB787 and the raw RNA-seq reads are available in NCBI under BioProject number PRJNA713911.
