# Supplementary material for: Analysis of Transcriptomic Response to SO2 by Oenococcus oeni Growing in Continuous Culture
Source: Microbiol Spectr. 2021 Oct 6;9(2):e01154-21. doi: 10.1128/Spectrum.01154-21 (PMC8510247; doi:10.1128/Spectrum.01154-21)
Supplement: Supplemental file 1 — Supplemental material. Download SPECTRUM01154-21_O.oeni_SO2_stress_Supplementary_tables.xlsx, XLSX file, 0.1 MB 10.1128/Spectrum.01154-21.2Supplemental file 2Supplemental material. Download SPECTRUM01154-21_Figure_S1.pdf, PDF file, 0.2 MB [file spectrum01154-21_figure_s1.pdf]

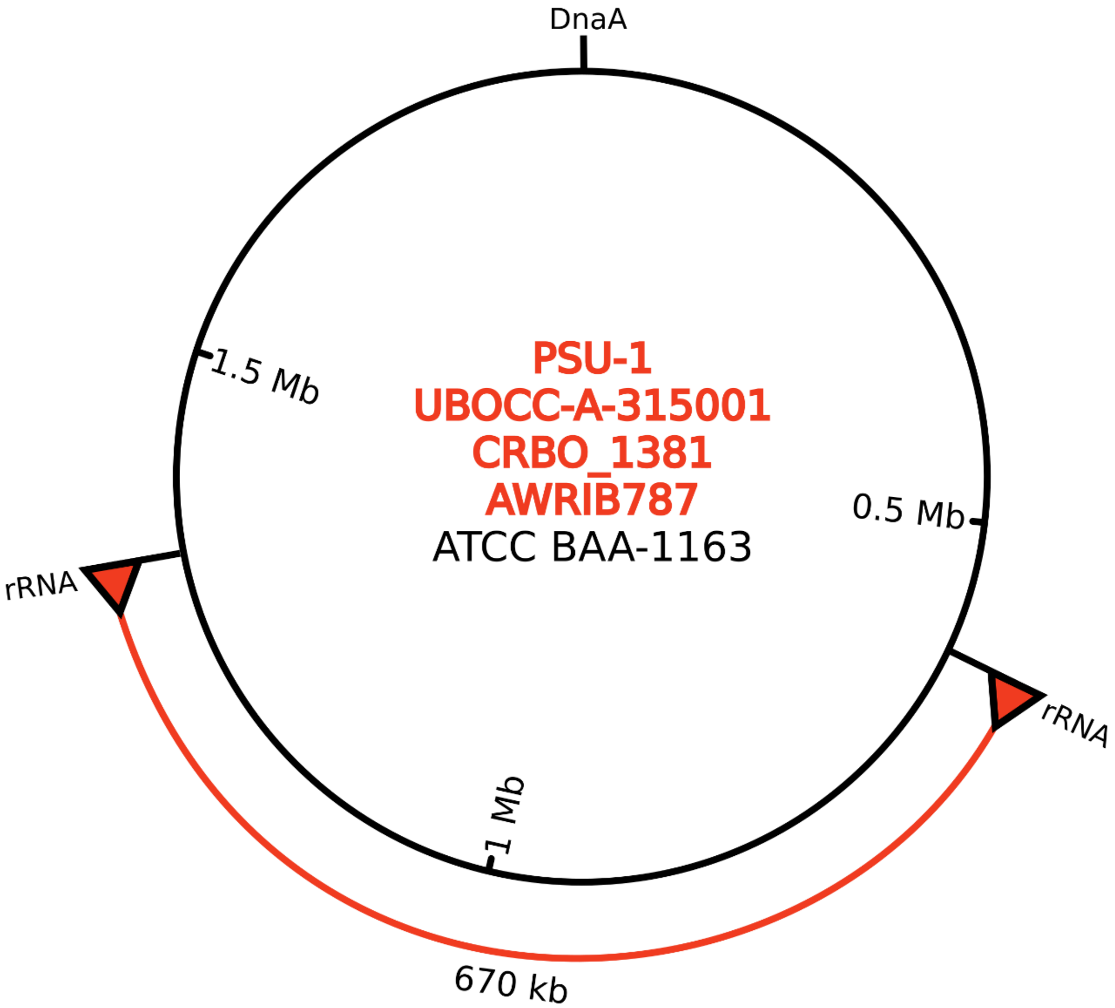

Figure S1. Chromosome synteny representation between publicly available single contig assemblies of *O. oeni* and the genome of strain AWRIB429. Orange line indicates a chromosomal inversion flanked by both rRNA operons.
